# Integrated Transcriptome‐Wide and miRNA Sequencing Analysis of Systemic Infection in Alzheimer's Disease Brain

**DOI:** 10.1002/alz70855_099626

**Published:** 2025-12-23

**Authors:** Giulia Pegoraro

**Affiliations:** ^1^ University of Exeter, Exeter, United Kingdom

## Abstract

**Background:**

Neuroinflammation is a key feature of Alzheimer's disease (AD), and episodes of systemic infection are known to exacerbate this process. Systemic inflammation triggers the production of pro‐inflammatory cytokines, which can activate microglia, the primary mediators of neuroinflammation in the brain. Evidence suggests that systemic infections not only increase the risk of developing dementia but also accelerate cognitive decline in AD patients. This study aims to explore the molecular mechanisms underlying the central nervous system response to systemic infections in AD by analysing changes in gene and microRNA (miRNA) expression.

**Method:**

RNA and miRNA sequencing was performed on prefrontal cortex tissue from 216 individuals, categorised by AD and infection status. Differential expression analysis was conducted using two‐way ANOVA on the two datasets. Weighted Gene Co‐expression Network Analysis (WGCNA) was applied to both gene and miRNA expression datasets to identify modules associated with AD and infection. Cell‐type enrichment analysis localized the expression of significant genes across brain cell types.

**Result:**

WGCNA identified a gene module associated with both infection and AD. Pathway enrichment analysis and functional module detection highlighted significant roles of viral budding and MAPK signaling, a critical pathway in neuroinflammation and immune response. Co‐expression analysis revealed *RIC8A*, a gene upregulated during infection in AD, as a potential regulator of cellular trafficking. Genes co‐expressed with *RIC8A* were enriched in Golgi vesicle transport, vesicle budding, and vesicle organisation pathways. Cell‐type enrichment showed that genes from the identified module were primarily expressed in endothelial cells, pericytes, and astrocytes, key components of the blood‐brain barrier (BBB). While specific miRNA findings remain to be established, initial sequencing results suggest potential miRNA involvement in regulating immune and vesicle transport pathways.

**Conclusion:**

These findings highlight the impact of systemic infections on neuroinflammatory responses in AD, mediated through immune‐related pathways and implicating BBB dysfunction. The enrichment of vesicle transport processes suggests infections may affect the AD brain via altered cellular trafficking. Future work will integrate RNA and miRNA sequencing data to elucidate whether miRNA dysregulation contributes to the observed differential expression of target genes.

